# A Toolbox for Controlling the Energetics and Localization of Electronic States in Self‐Assembled Organic Monolayers

**DOI:** 10.1002/advs.201400016

**Published:** 2015-02-18

**Authors:** Bernhard Kretz, David A. Egger, Egbert Zojer

**Affiliations:** ^1^ Institute of Solid State Physics, NAWI GrazGraz University of TechnologyPetersgasse 16A‐8010GrazAustria; ^2^ Department of Materials and InterfacesWeizmann Institute of ScienceRehovoth76100Israel

**Keywords:** collective electrostatics, density‐functional theory, interface design, monolayer quantum‐well, self‐assembled monolayer

## Abstract

Controlling the nature of the electronic states within organic layers holds the promise of truly molecular electronics. To achieve that we, here, develop a modular concept for a versatile tuning of electronic properties in organic monolayers and their interfaces. The suggested strategy relies on directly exploiting collective electrostatic effects, which emerge naturally in an ensemble of polar molecules. By means of quantum‐mechanical modeling we show that in this way monolayer‐based quantum‐cascades and quantum‐well structures can be realized, which allow a precise control of the local electronic structure and the localization of electronic states. Extending that concept, we furthermore discuss strategies for activating spin sensitivity in specific regions of an organic monolayer.

## 1 Introduction

With the ongoing drive to miniaturization, the dimensions of functional units in nanoscale materials become so small that their properties are essentially determined by interfaces. In many instances, a convenient way for tuning the characteristics of such interfaces is by using molecular (mono)layers. These can even adopt the role of functional elements in devices, for example, in highly efficient organic monolayer transistors^1^, ^2^ or as self‐assembled monolayer (SAM) based devices in the field of molecular electronics.^3^, ^4^, ^5^, ^6^, ^7^, ^8^, ^9^, ^10^, ^11^ The spatial localization and energetics of the electronic states in these layers play a crucial role, as the states serve as “channels” for the electrical current.^12^, ^13^ Therefore, the development of new concepts for wave‐function engineering is highly appealing, aiding both the improvement of existing device structures and the design of new device concepts.

In “classical” quantum‐devices based on inorganic semiconductors, such as quantum‐cascade lasers,^14^ the energetic positions and shapes of wave functions are tuned through growing stacks of 2D quantum‐wells with controlled thicknesses and compositions. When using organic semiconductors, quantum wells have been realized by a successive evaporation of layers with a thickness of only a few nanometers.^15^, ^16^, ^17^, ^18^, ^19^ A controlled periodic variation of the properties of organic structures within 2D layers has been discussed in the context of covalent organic frameworks (i.e., 2D polymers),^20^, ^21^ where wave‐function localization can be efficiently tuned by the chemical nature of the used building blocks.^22^ Beyond that also for more complex (metal–organic) interfaces adsorbate‐induced 1D or 0D quantum‐confinement effects have been discussed recently.^23^, ^24^

**Figure 1:**
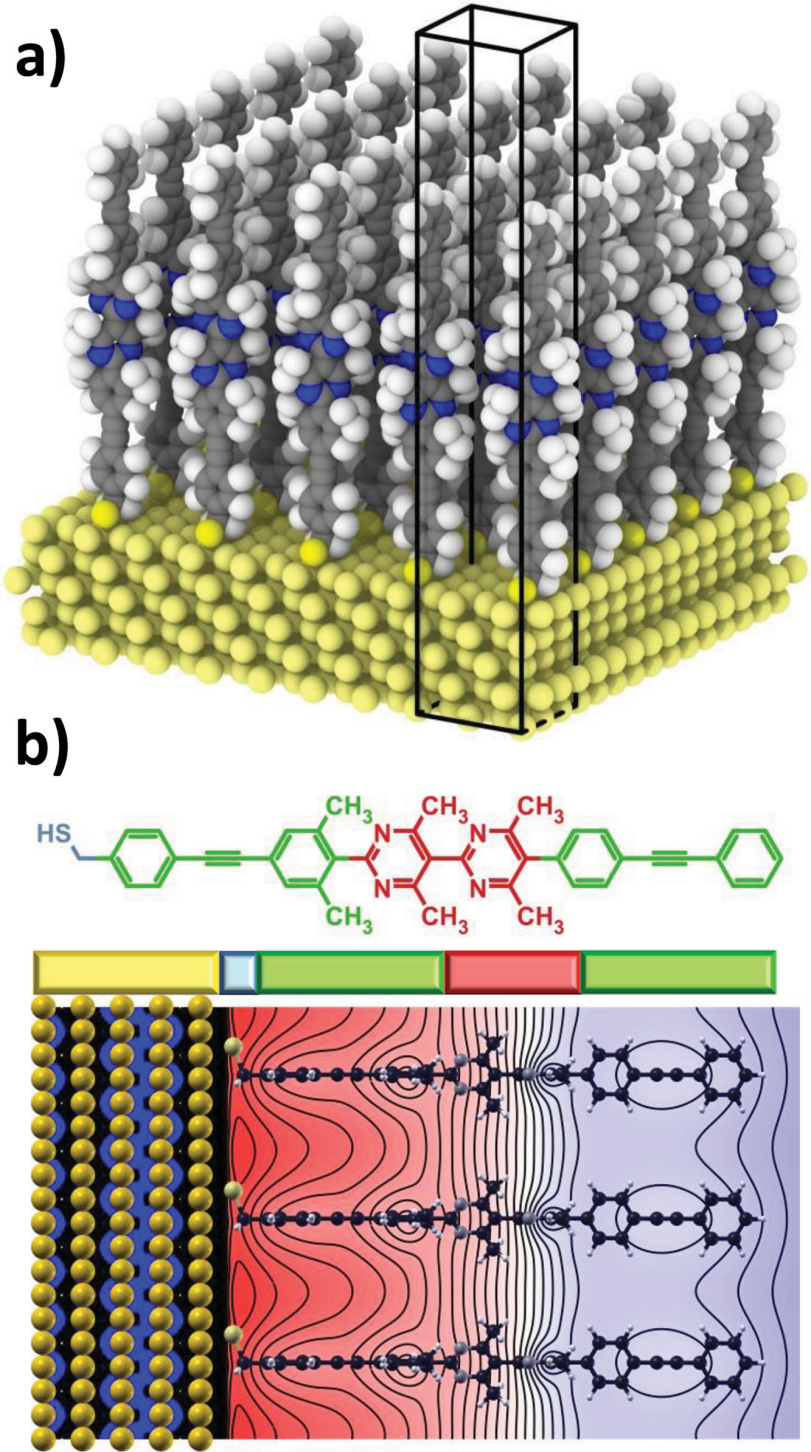
a) Arrangement of the molecules in a SAM consisting of a methylthiolate docking group and two 2‐phenylethynylbenzene segments separated by polar methylated bipyrimidine segments on a gold surface. b) Contour plot of the electron electrostatic energy in a plane close to the molecules (≈1.6 Å away from the hydrogen atoms to avoid oscillations near the nuclei); isolines are drawn every 0.1 eV and energy decreases from red to blue. The color bar on top of the contour plot sketches the extent of the substrate (yellow), the docking group (blue), the semiconducting (green), and the polar (red) segments. The chemical structure of the molecule is also shown prior to bonding to the substrate (i.e., still with S‐H instead of S‐Au bonds).

In the present contribution we suggest a conceptually different approach, developing the concept of a powerful and versatile toolbox that consists of functional elements for the realization of organic SAMs in which the energetics and orbital localization can be efficiently tuned. As will be shown, well‐known electrostatic effects can exploited to design SAMs with unprecedented electronic properties. As an exemplary case for our discussion we chose conjugated thiols that can be covalently bonded to Au surfaces (see **Figure** **1**a). We conceive a modular design strategy building the monolayers from individual functional parts. Our quantum‐mechanical calculations show that the resulting material systems display unprecedented properties, in particular regarding the energetics and localization of electronic levels; intriguing spin properties can also be realized.

## 2 Results

### 2.1 Collective Electrostatic Effects and the Concept of a Toolbox for Modular Interface Modifications

Central to the proposed approach are collective electrostatic effects that arise from dipolar elements built into the backbones of the SAM forming molecules^25^, ^26^ (and are not to be confused with cooperative effects impacting transport through monolayers via quantum–mechanical coupling).^7^, ^8^ The often very strong electrostatic effects are a consequence of the peculiar potential distribution created by the collective superposition of the fields of all molecular dipoles. This is best illustrated by plotting the electrostatic potential energy landscape of molecules with polar elements assembled as a 2D‐periodic monolayer. As an illustrative example we show in Figure 1b the situation for the prototypical model system also discussed later, which consists of two 2‐phenylethynylbenzene (tolan) sections separated by a polar, methylated bipyrimidine element. While for an isolated molecule in the gas‐phase all energy‐isolines have to be closed and, hence, no energy shift in regions far from the molecule occurs, in the SAM a qualitatively different situation is encountered:^25^ One sees a clear decrease of the electrostatic energy along the SAM that is most pronounced in the region of the polar units (schematically indicated by a red bar in Figure 1b). This results in a significant energy shift between the semiconducting sections “left” and “right” of the polar segment (indicated by the two green bars).

Another important physical property of the 2D‐arranged dipoles is that the drop in the electrostatic energy, which is proportional to the dipole density, is well confined to the pyrimidine units; i.e., the semiconducting segments are not significantly affected by “stray‐fields.” This is again a consequence of monolayer electrostatics,^26^, ^27^, ^28^ as the decay‐length of the stray field generated by an array of dipoles is nearly an order of magnitude shorter than the inter‐dipole distance. The absence of these “stray fields” is best seen in the rightmost semiconducting regions, where the potential is essentially constant. Note that in the left semiconducting region (i.e., close to the metal–organic interface) a minor gradient prevails, as shown in Figure 1b. This energy gradient is caused by bonding‐induced charge rearrangements at the SAM‐metal interface (see Figure S1, Supporting Information) and is absent for the situation of a hypothetical free‐standing monolayer (see Figure S2, Supporting Information). It is also inconsequential for the fundamental electronic properties of the SAM discussed below.

**Figure 2:**
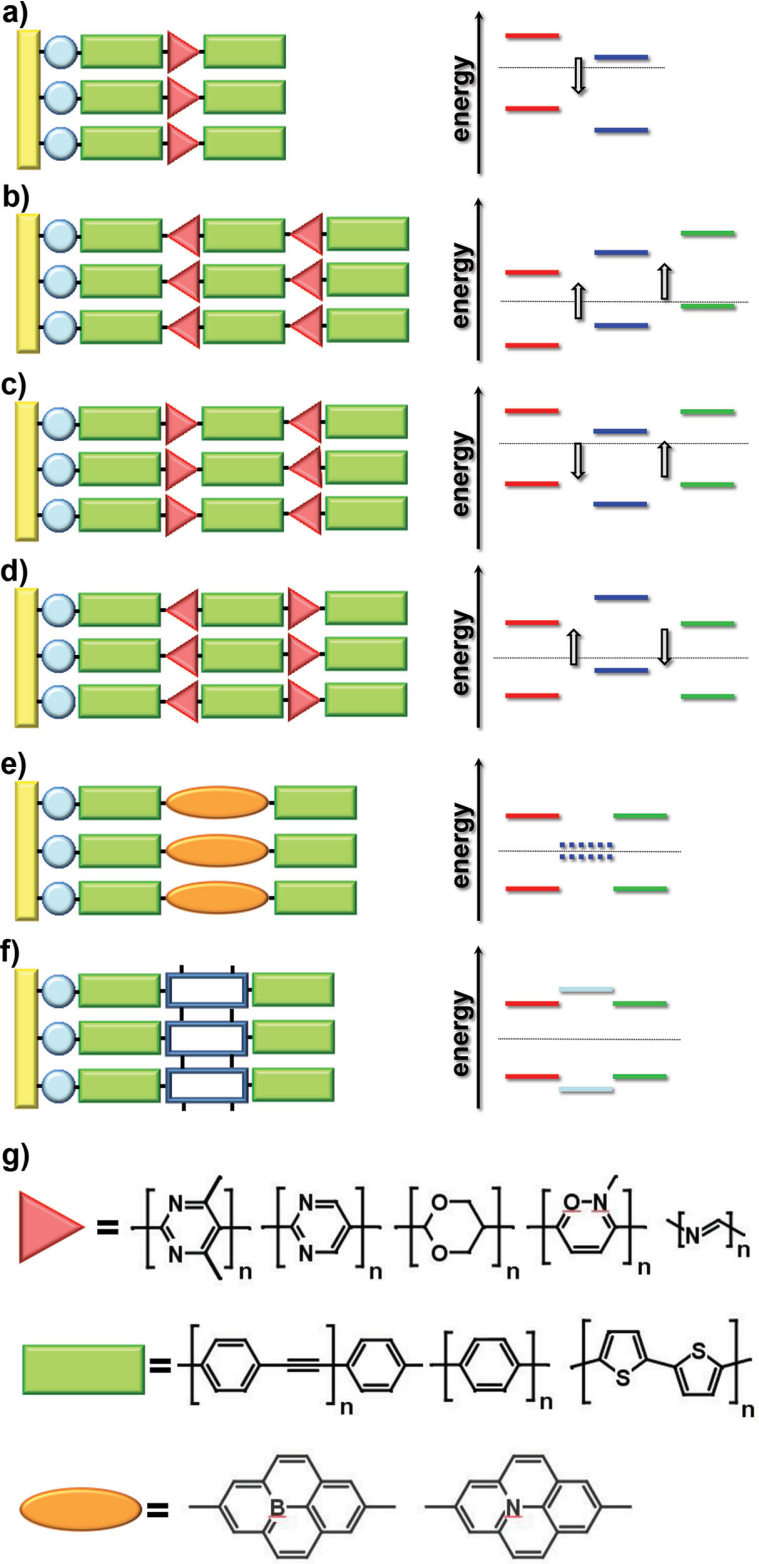
The concept of a “modular interface‐toolbox:” a–f) Left panels: Schematic structures of SAMs that can be realized combining the proposed elements of an “interface lego” toolbox. These comprise polar (red triangles), semiconducting (green rectangles), radical (orange ellipses), and H‐bonding elements (open blue rectangles), as well as suitable docking groups (blue discs) to bind the molecules to a substrate (yellow upright‐standing rectangle). a) This serves as the prototypical model system for the following discussion, in which we also present b) a quantum cascade, c,d) quantum‐wells, and e) a SAM containing a spin‐polarized segment. f) This serves as a typical example for a layer stabilized by bridging elements. a)–f) Right panels: Sketch of expected electronic structure of the SAMs realized by combining the aforementioned functional elements; the horizontal lines represent the frontier levels (local highest occupied and lowest unoccupied orbitals, respectively) in the individual segments, the thin dotted line designates the position of the Fermi‐level, E_F_. The vertical arrows indicate shifts in the electrostatic energy due to the inclusion of polar segments. The thick blue dotted lines in e) designate the spin–split spin‐up and spin‐down states around *E*_F_ in the radical segment. g) Possible examples for polar, semiconducting, and radical elements. For a discussion of docking and linking groups see main text.

The above discussed collective effects dominate work‐function modifications and level alignment in tail‐group substituted SAMs.^25^, ^28^, ^29^, ^30^, ^31^, ^32^, ^33^, ^34^, ^35^ They can also cause more exotic effects, such as the collectively induced quantum‐confined Stark effect in polar monolayers.^36^ Here, we intend to exploit them for locally manipulating the electrostatic potential, in this way shifting the energy levels in the semiconducting segments of a SAM and ultimately realizing SAMs with “user‐defined” properties (see **Figure** **2**). This approach goes far beyond previous efforts^36^ in that it directly utilizes emerging collective electrostatic effects in the design of complex, functional organic semiconducting layers.

To realize such functional layers, the polar elements (red triangles in Figure 2 with the orientation indicating the dipole direction) need to be combined with anchoring groups (blue discs), and semiconducting sections (green rectangles) that together form the first elements of a modular toolbox for controlling the electronic properties of monolayers. The combination of these elements already allows the realization of various functional structures with some straightforward examples shown in Figures 2a–d. The left panels sketch the schematic structure of the SAMs, while the right panels display the expected electronic properties derived from the collective electrostatic effects discussed above. The most straightforward and for the present study prototypical model structure is that shown in Figure 2a (with the system shown in Figure 1 being a possible realization of this structure). Following our above discussion, the collective electrostatic effects associated with the central polar element are expected to energetically shift the electronic states in the two semiconducting segments (cf. Figure 1b), as schematically indicated in the right panel of Figure 2a. Reversing the polarity of the polar elements is expected to also change the direction of this shift. When combining two (or more) polar units much more complex structures can be realized. For example, for polar units pointing in the same direction, quantum cascades can be realized (Figure 2b). In those, a “stepped” arrangement of the electronic states on the energy scale is expected (right panel of Figure 2b). Furthermore, when combining the same functional elements but reversing the orientation of either the first or the second polar unit, quantum‐well like structures are obtained, in which either the electrons (Figure 2c) or holes (Figure 2d) are confined to the central semiconducting sections.

Examples for possible chemical structures that serve as semiconducting and polar units are contained in Figure 2g. They comprise conjugated and nonconjugated polar elements that can be incorporated into the molecular backbones, as well as exemplary semiconducting sections. We did not include examples for linkers in Figure 2, as their nature very much depends on the used substrate.^25^

In addition to the functional elements discussed above, one can also conceive units consisting of organic radicals^37^, ^38^ with spin‐polarized electronic states close to the Fermi‐level (orange ellipses in Figure 2) or nonconjugated segments that electronically decouple parts of the SAM and are otherwise inactive (e.g., cyclohexane). Also elements that stabilize the structure of the monolayers, e.g., through hydrogen bonds^11^, ^39^ (indicated by dark‐blue open rectangles) are appealing. They will be important for more extended structures, as beyond a certain number of polar units, the bonding of the anchoring group to the substrate alone will no longer suffice to overcome dipole–dipole repulsion in ordered monolayers. Possible examples and the role of collective effects in such elements will be discussed towards the end of the paper.

By suggesting such a versatile toolbox, we intend to pave the way to a knowledge driven design of various classes of novel monolayer structures, with the examples of monolayer quantum cascades and quantum wells already mentioned and discussed in more detail below. To validate the conceptual idea outlined above, we studied the monolayers depicted schematically in Figure 2a–e by means of density‐functional theory (DFT) applying periodic boundary conditions and the repeated‐slab approach using the VASP^40^ (Vienna Ab initio Simulation Package) code (see Section 4).

### 2.2 Prototypical SAM

**Figure 3:**
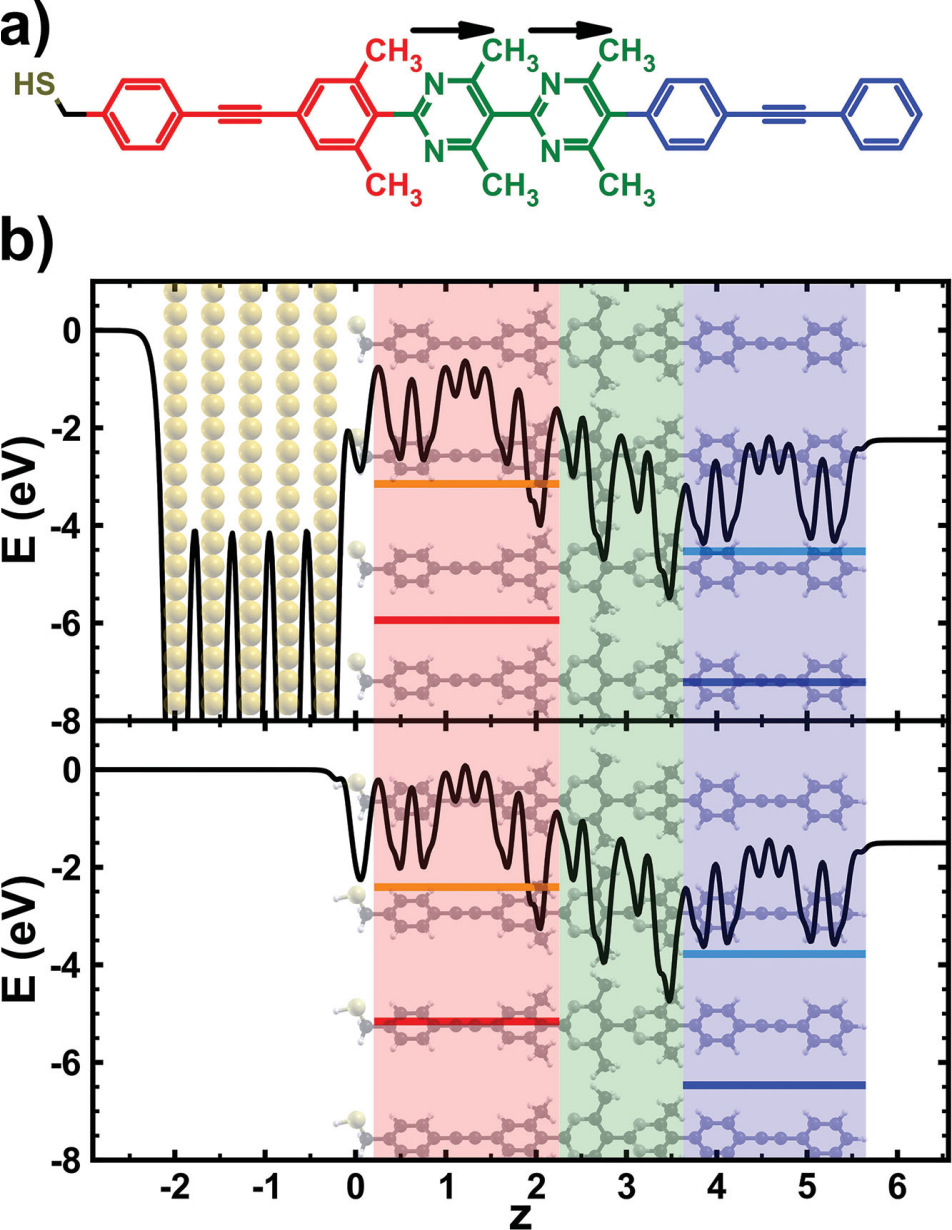
a) Schematics of the model molecule; different colors indicate the different units and the small arrows denote the direction of the dipole moments of the pyrimidine rings. b) Plane‐averaged electron electrostatic energy of the SAM on gold (upper plot) and the free‐standing monolayer (lower plot). The horizontal lines indicate the highest occupied substrate‐side localized orbital (red), the lowest unoccupied substrate‐side localized orbital (orange), the highest occupied vacuum‐side localized orbital (blue), and the lowest unoccupied vacuum‐side localized orbital (light blue).

For the molecular backbones discussed in the following examples, we picked the first structure contained in Figure 2g with *n* = 1 as the semiconducting element (i.e., using 2‐phenylethynylbenzene), see **Figure** **3**a. For the dipolar section substituted bypirimidine (*n* = 2) has been chosen, as pyrimidine‐containing systems have large dipole moments,^36^ allow for large work‐function changes,^31^ can be well integrated into conjugated backbones, and have been successfully synthesized and studied experimentally as potential molecular rectifiers.^41^, ^42^, ^43^ For the most simple systems discussed in the following we use a structure inspired by SAMs studied already experimentally to show the feasibility of the suggested approach. For the present purposes, we replaced pyrimidines by their methyl‐substituted analogues, as the twist induced by the substituents reduces conjugation between the pyrimidines preventing a (partial) delocalization of the lowest unoccupied orbital (LUMO) from the semiconductor segments onto the otherwise very low‐lying oligo‐pyrimidine LUMO. Additionally, the methyl substitution increases the dipole moment per repeat unit und, thus, boosts the net effect of the dipoles. To anchor the SAMs to a Au substrate, we used methylene thiolate, where the methylene serves to decouple the π‐system of the SAM from the extended electronic states in the Au substrate (cf., Figure 3a).^44^ For the present proof of principles simulations, we assembled the molecules in a comparatively large unit cell (3 × 2√3) in an upright‐standing position (for further details see Section 4; note that a significant tilt of the molecule would reduce the net effect as for the above‐discussed collective effects only the dipole moment perpendicular to the substrate surface counts).

The above choice leads to the structure shown in Figure 1a for the most simple prototypical SAM that is used to demonstrate the above concept (cf., Figure 2a). The plane‐averaged potential energy (Figure 3b) clearly displays the energetic shift between the left (red) and right (blue) semiconducting sections due to the collective action of the embedded dipoles. The net effect is virtually the same for the SAM on gold displayed in the upper panel and the (hypothetical) free‐standing layer shown in the lower panel. As a consequence of the massive potential energy gradient^36^ in the central (bipyrimidine) section of the SAM (green), which is consistent with the contour plot of the energy in Figure 1b, also the frontier orbitals localized on the left and right semiconducting unit are shifted significantly with respect to each other. The net shift amounts to −1.28 eV (−1.32 eV) for the highest occupied and −1.38 eV (−1.37 eV) for the lowest unoccupied states in the metal‐bonded SAM (respectively, free standing monolayer). Importantly, these shifts are fully consistent with the above‐discussed expectation for a system combining two semiconducting and one polar element from the above modular toolbox concept. Interestingly, indications for such an electrostatically induced shift due to embedding a dipolar segment in the backbone of a SAM‐forming molecule have indeed been observed for alkylthiolate SAMs containing an ester group.^45^ There, binding energies for the C1s features arising from the carbon atoms above, respectively, below the dipolar esters were found to be shifted by 0.8 eV with respect to each other by high‐resolution X‐ray photoelectron spectroscopy.

**Figure 4:**
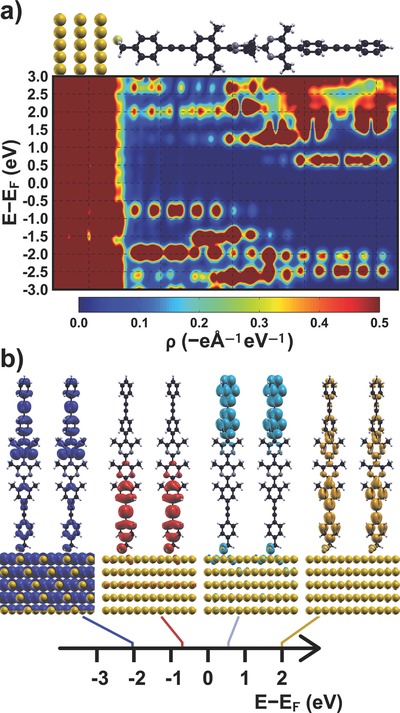
a) Contour map of the LDOS along the molecular backbone for the model SAM on gold obtained by integrating the LDOS in energy windows of 0.1 eV over the plane parallel to the Au surface within a unit cell (for a plot for the free‐standing monolayer see Figure S4, Supporting Information). b) Isodensity plots of the charge densities associated with the highest occupied and lowest unoccupied states within the semiconducting units of the model SAM on Au (the scale at the bottom of the plots gives the energies of the states relative to the Fermi‐level).

Beyond controlling the energies of the electronic levels, the localization of the relevant frontier states is another crucial aspect in the design of interfacial quantum‐structures. We visualize the localization of the electronic states using a local density of states (LDOS) contour map, which displays the charge density as a function of energy (on the vertical axis) and position along the molecular backbone (horizontal axis, see **Figure** **4**a).^46^ Clearly, the highest occupied state is confined to the left semiconducting unit (0.8 eV below *E*_F_) and the lowest unoccupied states on the right one (0.6 eV above *E*_F_). This strong localization is also well visible in the isodensity plots of the LDOS at the specific energies corresponding to the frontier states (central panels of Figure 4b). It is also recovered when projecting the density of states onto the three segments of the SAM (see Figure S3, Supporting Information). The highest occupied π‐state localized on the right semiconducting unit is found at −2.1 eV below *E*_F_, resulting in a “local” gap of 2.7 eV on that unit, which is by 1.3 eV larger than the “global” gap of 1.4 eV separating the overall highest occupied and lowest occupied states (for their nature compare results for the isolated molecule contained in Figure S5, Supporting Information). An equivalent situation is found for the right semiconducting segment. The change in the electrostatic environment of each segment of the monolayer and the associated shifts and localization of electronic states directly arises from the collective interaction of the embedded polar segments within the mono­layer. Hence, it is not surprising that in the limit of very low coverage as well as for the isolated molecule, where collective effects vanish, the asymmetry between the left and right semiconducting units are reduced by a factor of ≈2.6 (see Figures S5 and S6, Supporting Information).

From the results discussed so far, there is no qualitative difference between metal‐bonded and free‐standing monolayers in the effects we have observed, i.e., as far as state localization and energetic shifts are concerned. It should, however, be noted that when further increasing the number of dipolar units, the system enters the regime of Fermi‐level pinning.^47^, ^48^ The (globally) lowest unoccupied state or highest occupied state (depending on the dipole orientation) energetically approaches the Fermi‐level, triggering a compensating polarization of the SAM.^49^ As the occurrence of Fermi‐level pinning is determined by the relative energetic positions of *E*_F_ and the lowest occupied and unoccupied states in the SAM, the extent to which it affects the electronic structure of an interface is determined by the used metal substrate and will in most cases be absent for dielectric substrates, which are also of significant interest for SAMs with user‐defined properties.^1^ Thus, to avoid interference from a specific substrate, which is not the scope of our present work and highly complex in its own right, we will in the following focus on the properties of isolated monolayers disregarding the interaction with a specific surface. In fact, for all interfaces studied here (i.e., SAMs thiolate‐bonded to a Au (111) surface) only in the case of the cascade Fermi‐level pinning occurs limiting the total potential drop over the adsorbate layer.

### 2.3 Cascade

**Figure 5:**
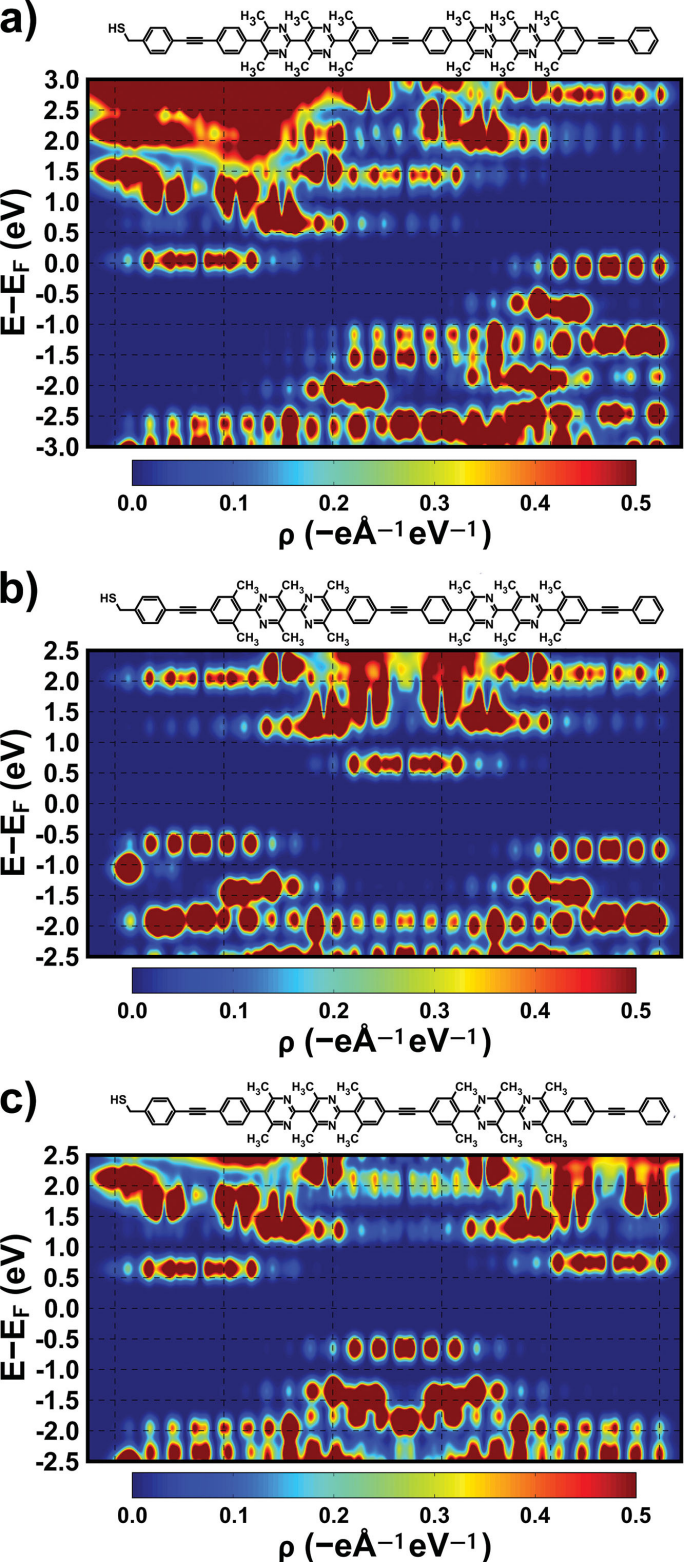
Contour map of the LDOS along the molecular backbone for free‐standing monolayers representing a) a quantum cascade, b) a quantum well for electrons, and c) a quantum well for holes. The energies are given relative to the Fermi‐level, E_F_, set to the middle of the energy gap.

The above‐discussed model system could, for example, serve as an interesting device for separating excitons into electrons and holes. By increasing the number of repeat units (i.e., polar plus semiconducting sections), even quantum‐cascades can be realized in the spirit of Figure 2b. A possible system in which this achieved is shown in **Figure** **5**a. Note that here we chose to portray the situation with inverted pyrimidine orientation to illustrate that our concept works independent of the dipole polarity. The energetic staircases for electrons and holes in the system are clearly visible with the energetic offsets between the left and central and the central and right semiconducting elements amounting to ≈1.2–1.5 eV. This is reminiscent of the “Stark‐ladder” phenomenon in the context of inorganic semiconductors, with the notable difference that no external field is applied in the present case and that the potential gradient is present only between the semiconducting units. Another interesting observation is that the global gap closes (i.e., the global highest occupied molecular orbital (HOMO) on the leftmost and the global LUMO at the rightmost semiconducting segments are at the same energy), while the local gap in each semiconducting unit is the same as for the reference system.^50^ The reason for the gap‐closure is that the combined energy shifts due to the two parallel 2D periodic bipyrimidine sheets are larger than the DFT‐calculated gap (see Section 4) of the 2‐phenylethynylbenzene semiconducting units.

### 2.4 Quantum Wells

Following the general layout of the quantum‐cascade structure, but flipping the orientation of one of the involved dipolar segments, the quantum‐well like structures presaged in Figure 2c,d can be realized. Our results for the electronic structure of these systems, displayed again as LDOS contour maps in Figure 5b,c, clearly show that following this design principle either the lowest unoccupied (“electron well”) or the highest occupied (“hole well”) states can be confined to the central semiconducting units of the SAM.

### 2.5 Spin‐Polarized Monolayers

**Figure 6:**
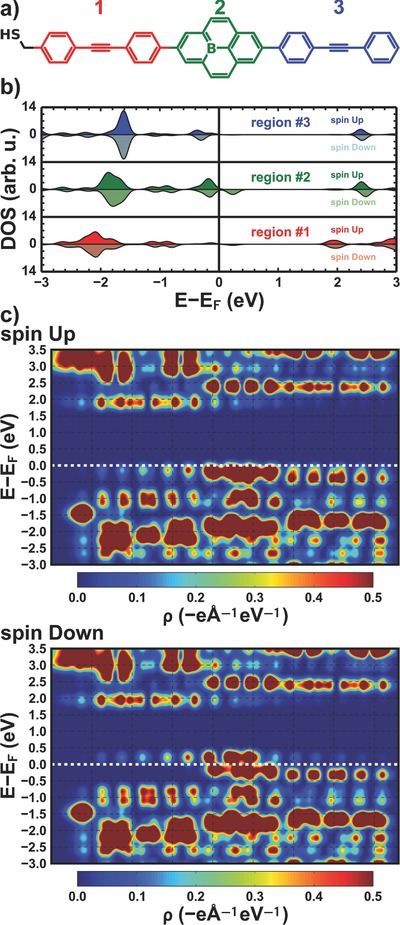
a) Chemical structure of a molecule forming a locally spin‐polarized SAM. b) Density of States of the corresponding free‐standing monolayer projected onto the three regions of the SAMs (#1 and #3 are semiconducting segments and #2 the neutral radical). The respective LDOS contour maps along the molecular backbone for the free‐standing monolayers for are shown in c) for the spin‐up and in d) for the spin‐down channel.

To realize a locally spin‐polarized monolayer the next element of the toolbox proposed in Figure 2, namely a molecular radical with an odd number of electrons needs to be included. A relatively simple system containing in addition to the radical segment (B‐substituted pyrene) only semiconducting units (2‐phenylethynylbenzene) is shown in **Figure** **6**a. Polar sections are avoided in this prototypical example for the sake of clarity.

The spin‐polarized nature of the electronic states in the B‐substituted pyrene segment (green) is clearly visible in the density of states projected onto the individual segments of the SAM shown in Figure 6b: Due to relatively close‐lying occupied (and unoccupied) frontier states in pyrene and the coupling between the pyrene‐segment and the semiconducting units, there are several bands in the vicinity of the Fermi‐level.^51^ For the spin‐up channel, these involve only states that are occupied, while for the spin down channel one of the bands comes to lie above *E*_F_. This local spin‐polarization is also clearly visible in the LDOS contour maps in Figure 6c (spin up channel) and Figure 6d (spin‐down channel). Such SAMs are of potential interest for spintronic applications.^52^, ^53^, ^54^, ^55^, ^56^, ^57^, ^58^ Combining these spin‐selective elements with other functional, e.g., polar units could even open up the way for entirely new applications, such as, magnetic molecular quantum wells. In this context it should be mentioned that the calculated amount of spin‐splitting between α‐ and β‐states depends on the employed xc‐functional.^59^ In fact, our results obtained with the PBE functional serve as a lower limit to the actual splitting.

### 2.6 SAMs Stabilized by Linkers

As an appealing outlook for future efforts, the development of linker elements stabilizing SAMs (e.g., via H‐bonds) is proposed. Designing the necessary molecules represents a clearly more involved task, as it inherently requires a spatial extent of functional elements that is compatible with inter‐molecular distances to be able to form inter‐molecular bonds. The inter‐molecular distances are in turn determined by the immediate substrate–organic interface (i.e., by the distance of the docking sites on the specific substrate used). Thus, while H‐bonded SAMs have been discussed in a few instances in the literature,^39^, ^60^ the detailed discussion of systems fully compatible with the functional elements discussed above goes clearly beyond the scope of the present paper. Potential starting points for their development could be natural H‐bonded chromophores such as quinacridone. These are, in fact, particularly interesting as they can give rise to collective electronic (albeit not electrostatic) effects beyond the stabilizing functionality. This has been discussed recently, e.g., for quinacridone films in the context of organic thin‐film transistors.^62^ The design of systems building on such H‐bonded chromophors bearing appropriate docking groups and a periodicity compatible with the docking sites of the substrate will, however, be a formidable task.

## 3 Conclusions

The above‐discussed examples show how the electronic structure of a SAM can be manipulated through collective electrostatic effects arising from the parallel alignment of dipolar building blocks. Going substantially beyond previous work, we have shown that combining them with semiconducting elements allows for the realization of quantum‐cascades or monolayer‐based quantum‐well structures. Furthermore, by including neutral radical segments into the SAMs, even locally spin‐polarized monolayers can be realized. While challenging to realize experimentally, these examples only serve as a first showcase hinting towards the potential of such an “electrostatic” toolbox for manipulating electronic properties of interfacial layers, which could be easily expanded by varying the band‐gaps of the semiconducting elements and the magnitudes of the potential shifts due to the dipolar units. Also alternative functional elements can be envisioned, such as chemical linkers or electronically decoupling sections. Thus, our results only mark the first step towards the development of a highly versatile and flexible toolbox.

## 4 Experimental Section

*Computational Approach*: All calculations discussed in this paper have been performed in the framework of DFT. The displayed results have been obtained using periodic boundary conditions and the PBE^63^ exchange‐correlation functional applying the VASP code.^40^ As has been shown before, in pyrimidine‐containing systems the frontier electronic states include close‐lying σ and π orbitals.^36^ A semilocal exchange‐correlation functional (such as PBE) leads to the more localized σ states lying spuriously too high in energy,^36^ which can be corrected with the use of a hybrid functional.^36^ While this in principle also applies to the systems studied in this work, this affects only the relative order of the orbitals within the shifting units, which is of no relevance for the localization effects and energy shifts in the semiconducting units that are in the focus of the present paper. To model the breaking of the translational symmetry by the surface the repeated‐slab approach has been used, where we represented the Au(111) surface by five layers of metal atoms. Periodic replicas of the slab are separated by an at least 40 Å wide vacuum gap containing a self‐consistently determined dipole layer.^64^ Typically, a 4 × 5 × 1 Monkhorst‐Pack type^65^ k‐point grid has been used; only when studying the radical‐containing SAM, where we chose a smaller unit cell (see below), a 8 × 5 × 1 grid has been applied. When available, soft projector augmented‐wave (PAW) potentials^66^, ^67^ were chosen (all used potentials are listed in Supplementary Table 1) and the cutoff‐energy for the basis set was set to 273.894 eV. Obtaining a reliable electronic structure of the SAMs was complicated by the very large aspect ratios of the unit cells, the sometimes occurring long‐range charge rearrangements between the two ends of the SAMs in conjunction with Fermi‐level pinning (especially for cascades), and the comparably small energetic separation between the unoccupied frontier states at which that pinning occurs (cf., Figure S5, Supporting Information). To overcome these problems it was necessary in the final self‐consistent field (SCF) calculation including the self‐consistently determined dipole layer (i) to use a comparably small Gaussian broadening of the electronic states of σ = 0.02 eV, (ii) to employ an additional support grid for the evaluation of the augmentation charges,^68^ and (iii) to perform the evaluation of the projection operators in real space.^68^ If instead more “conventional” settings were used in the calculations, a pronounced dependence of the obtained electronic structure, on, e.g., the position of the auxiliary dipole layer within the vacuum gap or the overall extent of the latter were observed (cf., Figure S8, Supporting Information). Energies of states localized on specific segments were determined from the maxima of the peaks of the highest occupied (lowest unoccupied) states of the DOS's projected onto individual segments of the SAM. The visualization of the data was done with the python‐library matplotlib^69^ and for the isodensity plots Xcrysden^70^ was used (isovalue of 0.06 eÅ^−3^ eV^−1^). The density of states was integrated in an energy window with a width of 0.1 eV around the peak positions.

*System Setup*: The monolayer structures were obtained using the following approach: Molecular geometries were first optimized using Gaussian03^71^ with the PBE^63^ functional and the 6‐31G* basis set and then arranged upright standing in a (3 × 2√3) surface unit‐cell. In the absence of information regarding the actual packing of the molecules on the surface, we chose this rather large unit cell to avoid spurious interactions between periodic replicas. Test calculations on SAMs with less bulky unsubstituted pyrimidine units showed qualitatively similar results, with the overall magnitude of the effect reduced due to the smaller dipole moments (vide supra) and the particularly low‐lying LUMO of fully conjugated pyrimidines strongly coupling to the lowest unoccupied states on the low‐energy side of the semiconducting section. The less bulky pyrene‐containing SAMs were arranged in a (√3 × 3) surface unit‐cell. To determine the binding site for the thiolates in all metal‐bonded SAMs, we performed a full geometry optimization of methylthiolate on Au(111), from which we deducted an adsorption site between the fcc hollow site and the bridge site. The relaxations of the top two metal layers that we received from this preoptimization were then adopted throughout all our simulations.

## Supporting information

As a service to our authors and readers, this journal provides supporting information supplied by the authors. Such materials are peer reviewed and may be re‐organized for online delivery, but are not copy‐edited or typeset. Technical support issues arising from supporting information (other than missing files) should be addressed to the authors.

Supplementary
