# Supplementary material for: A Toolbox for Controlling the Energetics and Localization of Electronic States in Self‐Assembled Organic Monolayers
Source: Adv Sci (Weinh). 2015 Feb 18;2(3):1400016. doi: 10.1002/advs.201400016 (PMC4973851; doi:10.1002/advs.201400016)
Supplement: Supplementary file 1 — Supplementary [file ADVS-2-0d-s001.pdf]

Copyright WILEY-VCH Verlag GmbH & Co. KGaA, 69469 Weinheim, Germany, 2015.

Supporting Information

for *Adv. Sci.,* DOI: 10.1002/advs.201400016

A Toolbox for Controlling the Energetics and Localization of Electronic States in Self-Assembled Organic Monolayers

*Bernhard Kretz, David A. Egger, and Egbert Zojer**

A Toolbox for Controlling the Energetics and Localization of Electronic States in Self-Assembled Organic Monolayers

*Bernhard Kretz†, David A. Egger, and Egbert**

Bernhard Kretz, Dr. David Egger, Prof. Egbert Zojer

Institute of Solid State Physics, NAWI Graz, Graz University of Technology, Petersgasse 16, A-8010 Graz, Austria.

E-mail: Egbert.zojer@tugraz.at

Dr. David Egger

Department of Materials and Interfaces, Weizmann Institute of Science, Rehovoth 76100, Israel.

Present Addresses

†Donostia International Physics Center (DIPC), Paseo Manuel de Lardizabal 4, E-20018 San Sebastiàn, Spain

1. Charge-rearrangements upon metal-SAM bond formation

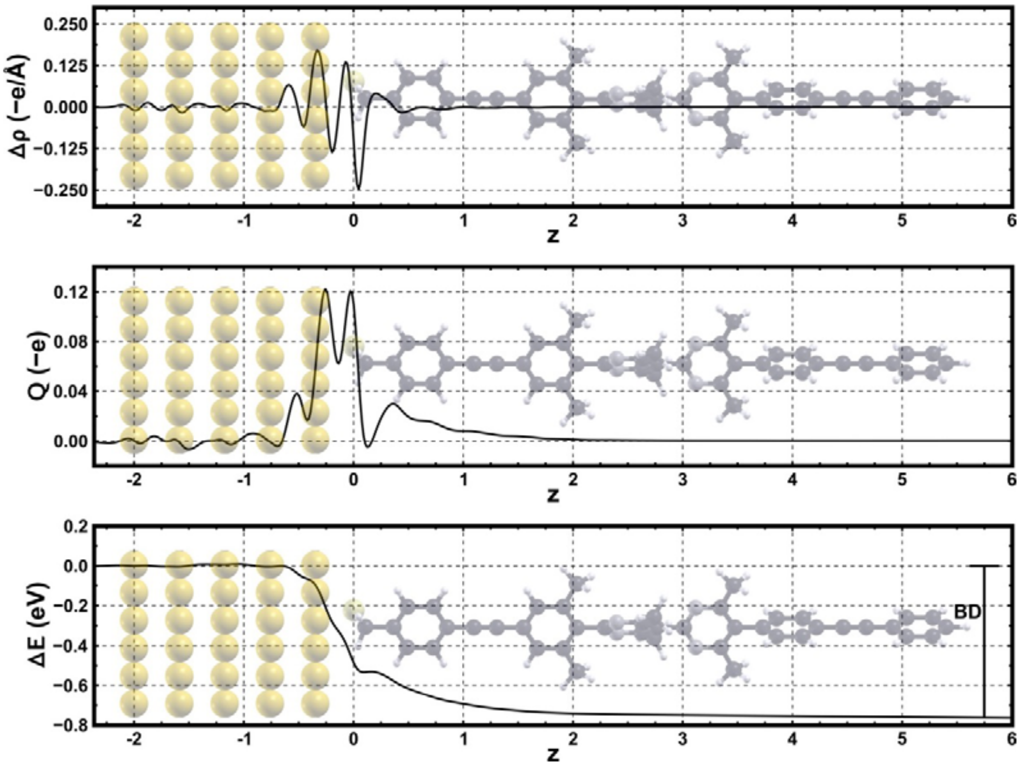

Figure S1. Top: Plane-integrated charge rearrangements in the model SAM upon replacing the S-H by a S-Au bond upon molecule to Au(111) bonding; center: cumulative charge rearrangements obtained by integrating Δρ over z. Q(z) describes the net charge transferred from right to left of a plane positioned at z; bottom: resulting change in the electrostatic energy of an electron as obtained from solving the Poisson equation. The majority of the charge rearrangements occur at the interface and result in an energy step of amount "BD".

2. Electrostatic energy in a free-standing monolayer

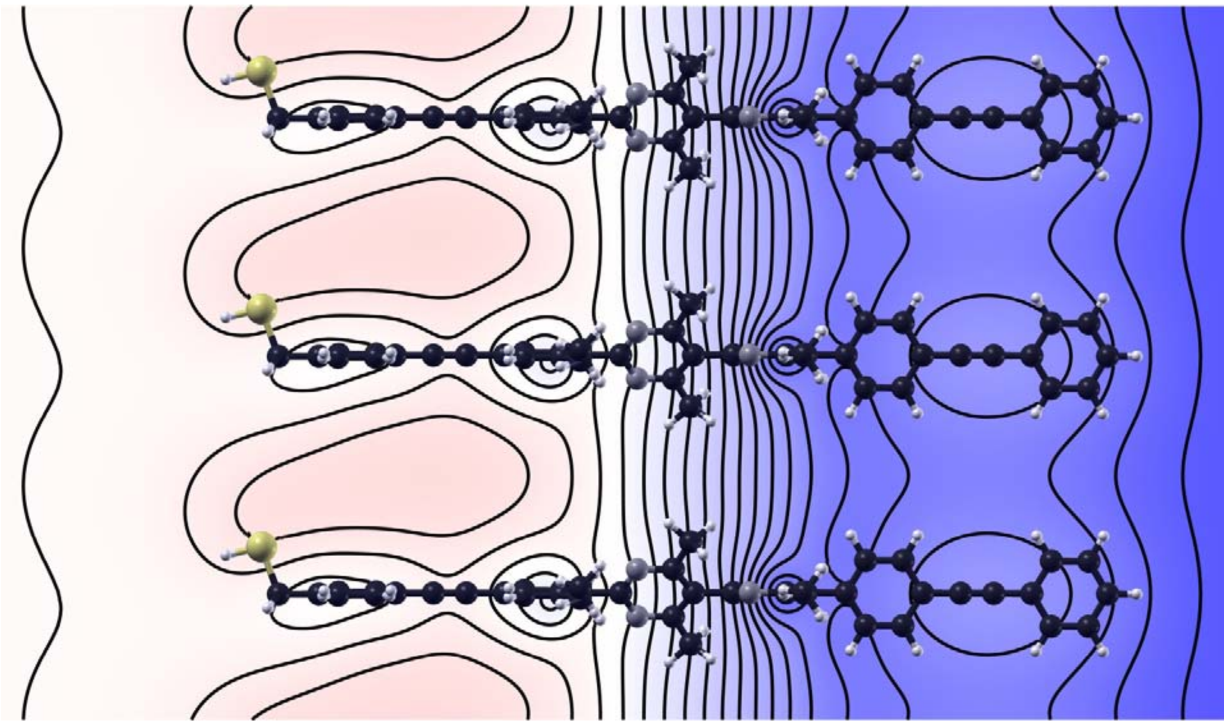

Figure S2: Contour plot of the electron electrostatic energy for a monolayer consisting of a methylthiolate group and two Tour-wire type segments separated by polar methylated bipyrimidine segments in a plane close to the molecules (approx. 1.6 Å away from the hydrogen atoms to avoid oscillations near the nuclei); isolines are drawn every 0.1 eV.

3. Density of states of the model SAM bonded to Au(111)

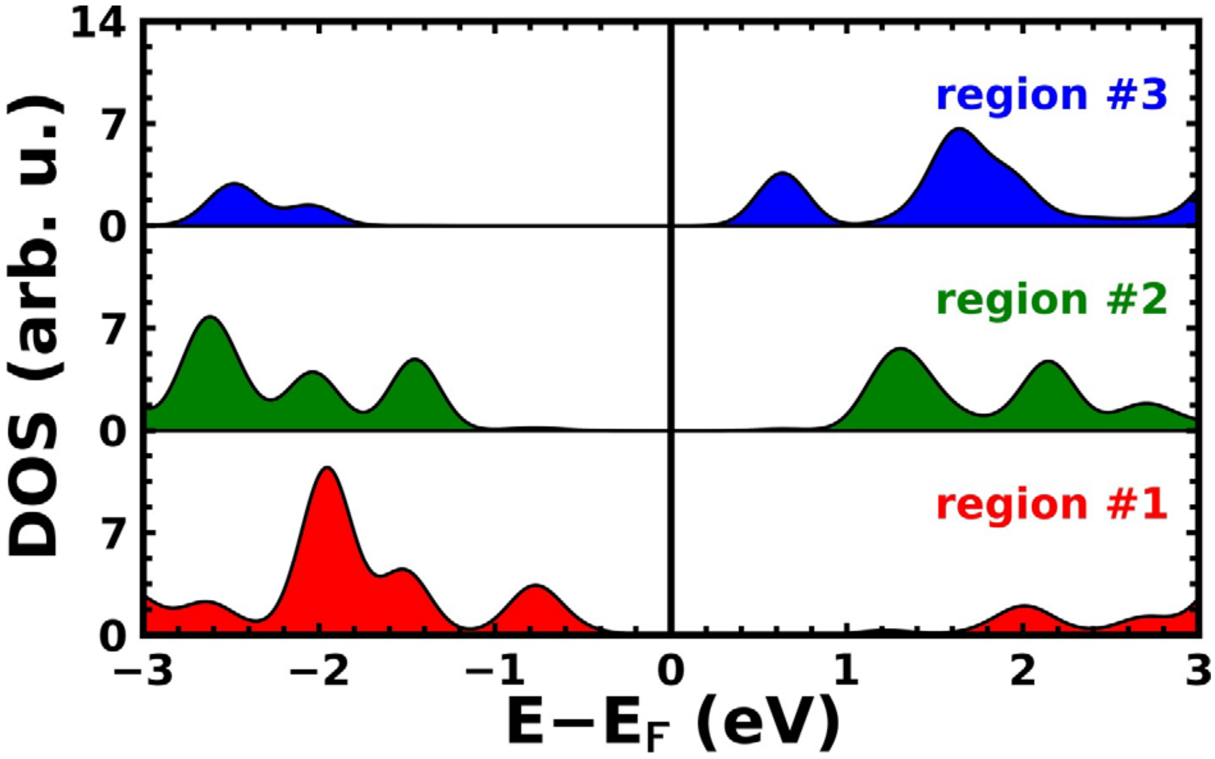

Figure S3: DOS of the model SAM projected onto the three units defined in Figure 3. The onset of Fermi-level pinning at the unoccupied states in unit 3 (closest to the metal substrate) is clearly visible.

4. LDOS contour map for free-standing model system

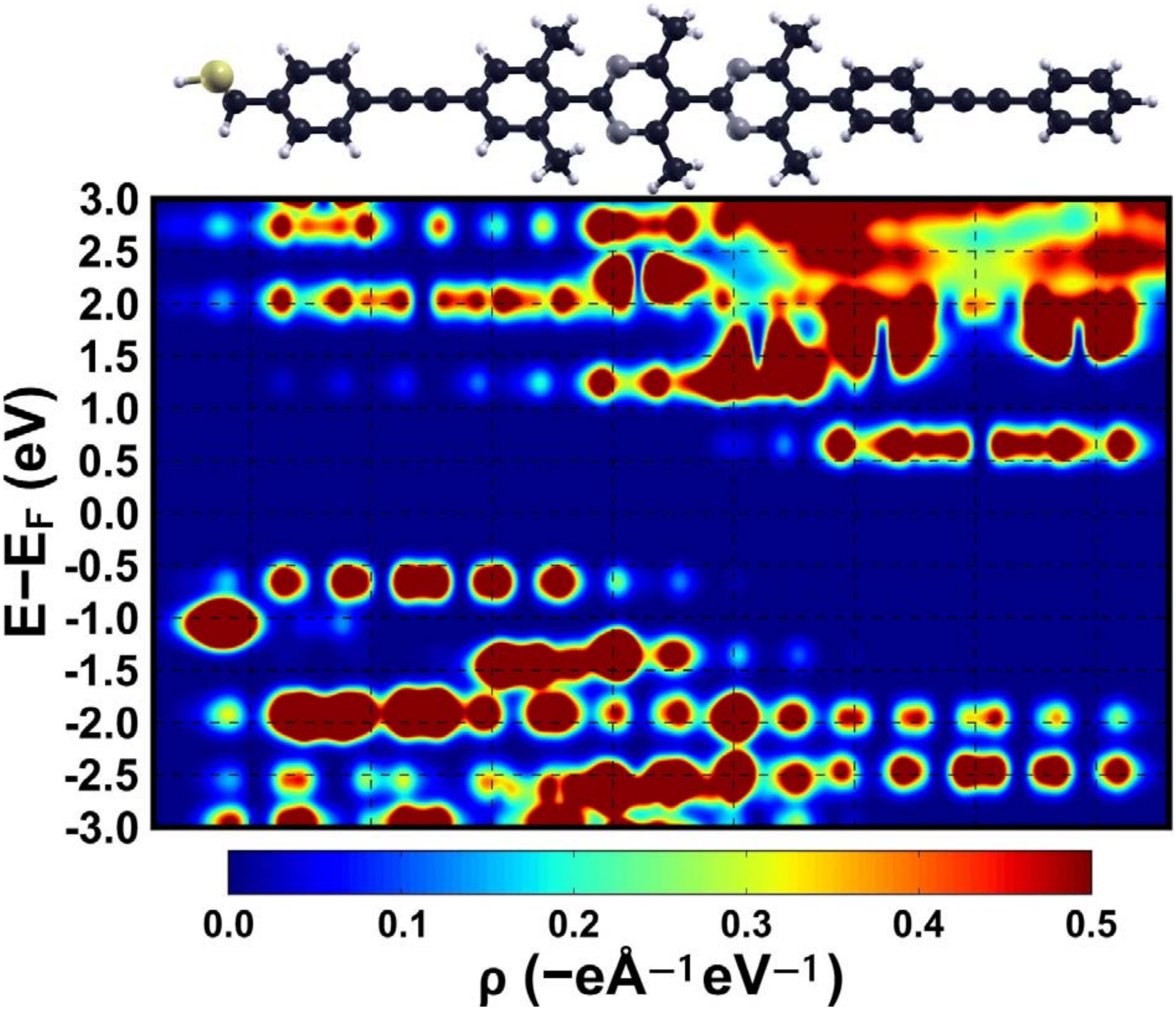

Figure S4: Contour map of the local density of states (LDOS) along the molecular backbone for the free-standing model SAM not bonded to gold obtained by integrating the LDOS in energy windows of 0.1 eV over the plane perpendicular to the SAM within a unit cell.

5. Electronic structure of the isolated molecule and low-coverage monolayer

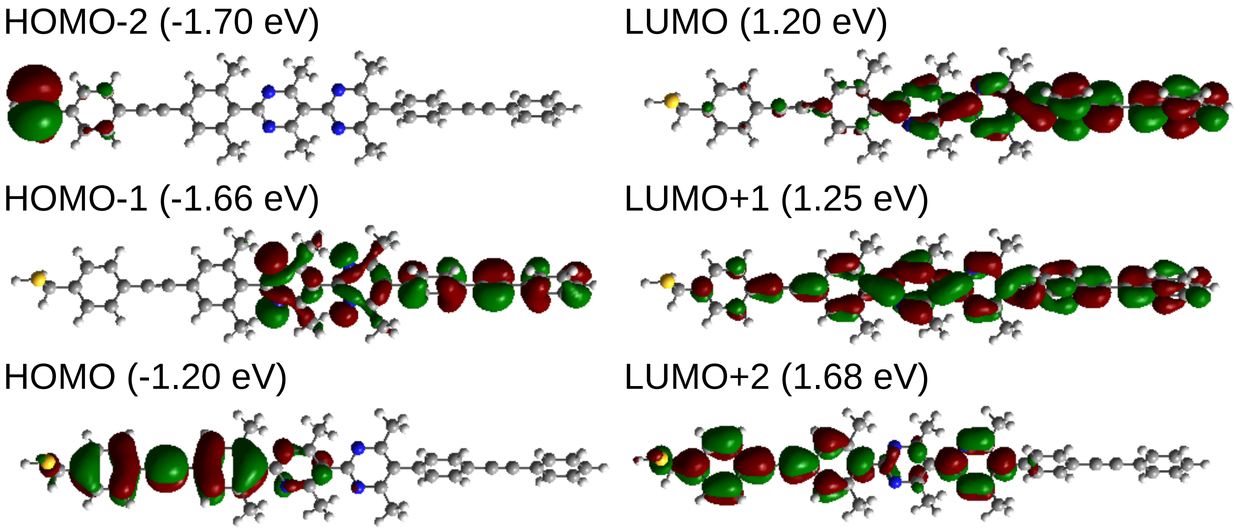

Figure S5: Isodensity plots and energies (relative to ½($\varepsilon_{LUMO}+\varepsilon_{HOMO}$) of the molecule forming the model SAM consisting of two 2-phenylethynylbenzene separated by a methyl-substituted bipyrimidine segment and bearing a thiol group. The used geometry was the same as in all other calculations presented in the main text. For the sake of comparability, the molecular orbital structure has been calculated using the PBE functional (and a 6-311+G(d,p)) basis set. For this calculation, Gaussian03 (i.e., an open boundary condition code) has been used to avoid any interference from neighboring unit cells. The “hybrid” σ/π HOMO-1 is a consequence of the very large twist angle of 84° between the 2-phenylethynylbenzene and the methyl-substituted bipyrimidine segments. Its occurrence is also triggered by the incorrect relative ordering of π- and σ-states in pyrimidines when using GGA functionals.[1] In fact, test calculations show that this effect is strongly reduced when applying a hybrid functional. What is also clearly visible is that in the absence of collective effects, the unoccupied states are no longer localized on the right semiconducting units owing to the low-lying π* states in the pyrimidines. The fact that the LUMO and LUMO+1 are so close in energy is attributed to the strongly reduced conjugation

resulting from the large twist between the upper doubly methyl-substituted pyrimidine and the upper semiconducting unit.

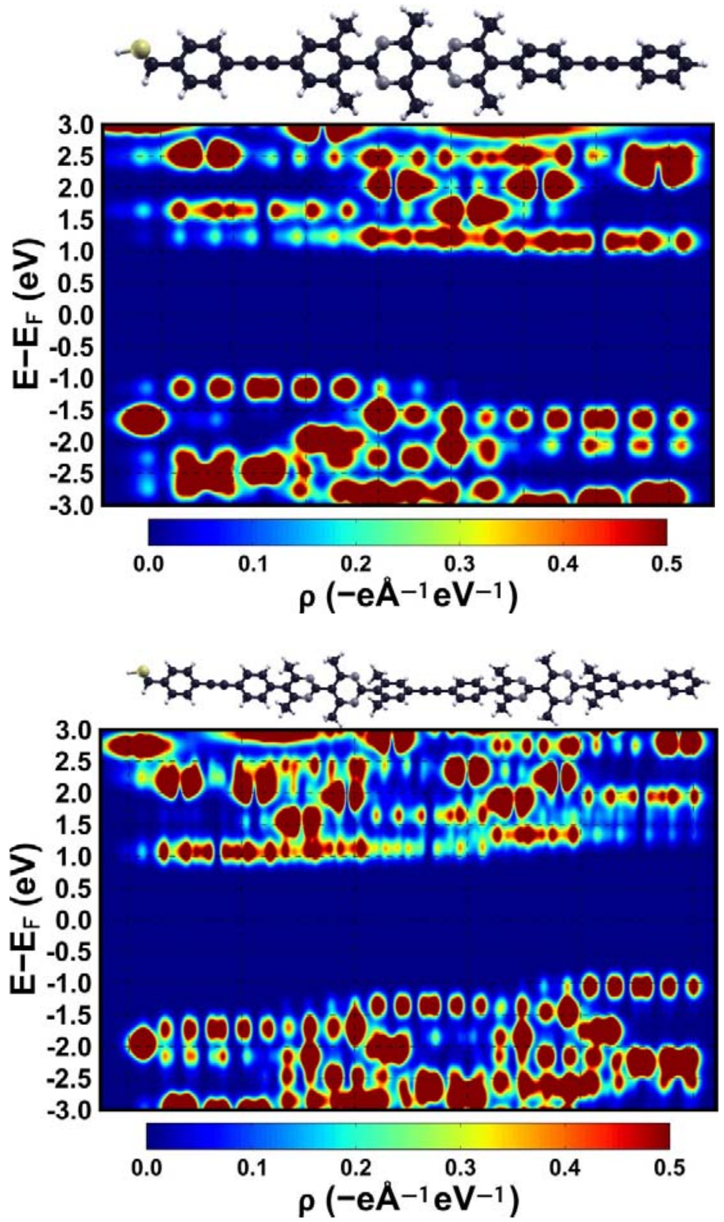

Figure S6: Top: Contour map of the local density of states (LDOS) along the molecular backbone for the free-standing model SAM at strongly reduced coverage. The quantity is

obtained by integrating the LDOS in energy windows of 0.1 eV over the plane perpendicular to the molecular axis. Bottom: equivalent plot for the molecule in the cascade. The very small energetic shift between neighboring semiconducting segments as compared to the situation in the monolayer (see Supplementary Figure 4 and Figure 3 in the main paper) due to the strongly reduced collective electrostatic effects is clearly visible. For the calculation a unit cell with a 40 Å x 40 Å base (and a 1 x 1 x 1 k-point mesh) have been used compared to the otherwise employed unit cell with a 8.86 Å x 10.23 Å base (corresponding to a 2x2√3 structure). Overall, the situation in the low-coverage SAM is strongly reminiscent of that in the isolated molecule (see next section.

6. Additional methodological details

Supplementary Table 1 | Overview of the used PAW potentials (the listed names correspond to the headers in the PAW potentials officially supplied with the VASP code):

Au	- Au 06Sep2000
S	- S 17Jan2003
N	- N_s 07Sep2000
C	- C_s 06Sep2000
B	- B_s 22Jan2003
H	- H 15Jun2001

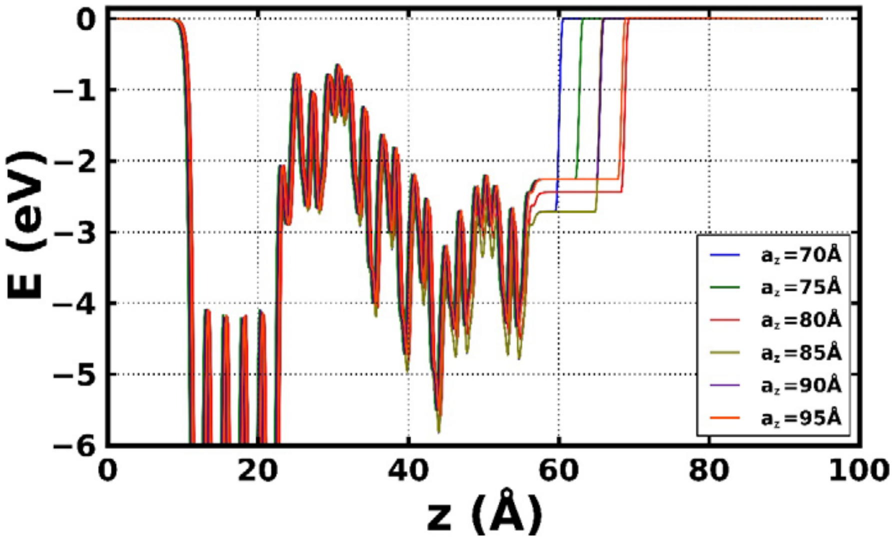

Figure S7: Plane-averaged electron electrostatic energy of the SAM for different unit-cell heights (i.e., extents of the vacuum gap) when using conventional VASP settings and a cutoff energy of 273.894eV (i.e., when the accuracy is not increased and the convergence is not slowed down). One clearly sees that a non-systematic variation of the net-potential drop over the SAM is obtained arising from an incorrect/incomplete convergence.

1. Rissner, F. *et al.* Collectively Induced Quantum-Confined Stark Effect in Monolayers of Molecules Consisting of Polar Repeating Units. *J. Am. Chem. Soc.* **133,** 18634–18645 (2011).
